# Introduction of a multiplex amplicon sequencing assay to quantify DNA methylation in target cytosine markers underlying four selected epigenetic clocks

**DOI:** 10.1186/s13148-023-01545-2

**Published:** 2023-08-10

**Authors:** Ewelina Pośpiech, Aleksandra Pisarek, Joanna Rudnicka, Rezvan Noroozi, Michał Boroń, Aleksander Masny, Bożena Wysocka, Kamila Migacz-Gruszka, Dagmara Lisman, Paulina Pruszkowska-Przybylska, Magdalena Kobus, Maria Szargut, Joanna Dowejko, Kamila Stanisz, Julia Zacharczuk, Piotr Zieliński, Aneta Sitek, Andrzej Ossowski, Magdalena Spólnicka, Wojciech Branicki

**Affiliations:** 1 Malopolska Centre of Biotechnology, Jagiellonian University, Krakow, Poland; 2 Department of Forensic Genetics, Pomeranian Medical University in Szczecin, Szczecin, Poland; 3 Institute of Zoology and Biomedical Research, Faculty of Biology, Jagiellonian University, Krakow, Poland; 4 Doctoral School of Exact and Natural Sciences, Jagiellonian University, Krakow, Poland; 5 Central Forensic Laboratory of the Police, Warsaw, Poland; 6 Department of Dermatology, Collegium Medicum of the Jagiellonian University, Krakow, Poland; 7 Department of Anthropology, Faculty of Biology and Environmental Protection, University of Łódź, Łódź, Poland; 8 Institute of Biological Sciences, Faculty of Biology and Environmental Sciences, Cardinal Stefan Wyszynski University in Warsaw, Warsaw, Poland; 9 Institute of Environmental Sciences, Faculty of Biology, Jagiellonian University, Krakow, Poland; 10 Center for Forensic Science, University of Warsaw, Warsaw, Poland; 11 Institute of Forensic Research, Krakow, Poland

**Keywords:** DNA methylation analysis methods, High-throughput sequencing, Target enrichment protocols, Epigenetic age estimation, Pace of aging, Mortality risk score

## Abstract

**Background:**

DNA methylation analysis has proven to be a powerful tool for age assessment. However, the implementation of epigenetic age prediction in diagnostics or routine forensic casework requires appropriate laboratory methods. In this study, we aimed to compare the performance of large-scale DNA methylation analysis protocols that show promise in terms of accuracy, throughput, multiplexing capacity, and high sensitivity.

**Results:**

The protocols were designed to target a predefined panel of 161 genomic CG/CA sites from four known estimators of epigenetic age-related parameters, optimized and validated using artificially methylated controls or blood samples. We successfully targeted 96% of these loci using two enrichment protocols: Ion AmpliSeq™, an amplicon-based method integrated with Ion Torrent S5, and SureSelect^XT^ Methyl-Seq, a hybridization-based method followed by MiSeq FGx sequencing. Both protocols demonstrated high accuracy and robustness. Although hybridization assays have greater multiplexing capabilities, the best overall performance was observed for the amplicon-based protocol with the lowest variability in DNA methylation at 25 ng of starting DNA, mean observed marker coverage of ~ 6.7 k reads, and accuracy of methylation quantification with a mean absolute difference between observed and expected methylation beta value of 0.054. The Ion AmpliSeq method correlated strongly with genome-scale EPIC microarray data (*R* = 0.91) and showed superiority in terms of methylation measurement accuracy. Method-to-method bias was accounted for by the use of linear transformation, which provided a highly accurate prediction of calendar age with a mean absolute error of less than 5 years for the VISAGE and Hannum age clocks used. The pace of aging (PoAm) and the mortality risk score (MRS) estimators included in our panel represent next-generation clocks, were found to have low to moderate correlations with the VISAGE and Hannum models (*R* < 0.75), and thus may capture different aspects of epigenetic aging.

**Conclusions:**

We propose a laboratory tool that allows the quantification of DNA methylation in cytosines underlying four different clocks, thus providing broad information on epigenetic aging while maintaining a reasonable number of CpG markers, opening the way to a wide range of applications in forensics, medicine, and healthcare.

**Supplementary Information:**

The online version contains supplementary material available at 10.1186/s13148-023-01545-2.

## Background

DNA methylation associates with aging, and a growing body of research has discovered thousands of age-related CpG markers with predictive potential [1–6]. Analysis of DNA methylation markers, which are subject to inter-individual variability, allows determination of the individual rate of aging. Epigenetic age acceleration (EAA) reflects a deviation from chronological age, has been linked to age-related diseases, stress, and lifestyle-related risk factors, and is a powerful biomarker with potential applications in clinical trials, risk assessment, and prevention [7–10].

Epigenetic age estimation can provide valuable clues in forensic investigations by narrowing down the circle of suspects and speeding up the process of human identification [11, 12]. Age is also important in the process of data interpretation in the investigative genetic genealogy [13] and in forensic DNA phenotyping to improve genetic prediction of progressive appearance traits [14]. The use of DNA methylation analysis in forensics goes beyond age prediction and can be used, for example, to identify tissues or distinguish between identical twins [15].

DNA methylation quantification and data interpretation are considered demanding for routine laboratory implementation compared to standard human identification protocols involving microsatellite and SNP testing [15, 16]. Due to the reduced complexity and high degradation of bisulfite-treated DNA, the multiplexing capabilities and the sensitivity of the methods are limited [17]. In addition, because of the quantitative nature of DNA methylation analysis, there is a method-to-method bias that needs to be addressed appropriately when data from different sources are interpreted [18]. Epigenome-wide assays are commonly used in research to analyze DNA methylation, with the Infinium Human Methylation 450 K/EPIC microarray assays often considered the gold standard, but their utility in routine laboratories suffers from labor-intensive protocols, high input DNA requirements, and complex data handling [19]. Validated tools and protocols are needed to enable quick, easy, robust, and cost-effective analysis of a set of predefined DNA methylation markers in the target laboratory [20]. There is also a strong need to develop recommendations for a standard operating procedure for DNA methylation analysis and interpretation of the results obtained [16], and important ethical issues in forensic age prediction are widely discussed [21].

So far, the most commonly used technologies have been pyrosequencing [3, 22], minisequencing [23, 24], and EpiTyper [18, 25]. Although these methods have advantages, their multiplexing capabilities are limited. High-throughput DNA sequencing technology (HTS) may provide greater opportunities in this regard [19, 26–30]. Recently, the VISAGE consortium published two validated protocols for DNA methylation measurement at selected eight and five loci based on multiplex bisulfite PCR followed by sequencing on the MiSeq FGx platform [31–33]. The developed tests, in conjunction with mathematical models [33, 34], enable the estimation of chronological age from down to ~ 20 ng of DNA with an accuracy of mean absolute error (MAE) of < 4 years in selected somatic tissues and ~ 5 years in semen samples, respectively. Lower amounts of initial DNA have also been reported for bisulfite amplicon sequencing in other studies [28]. However, due to the in-house design of the PCR reaction used, multiplexing a larger number of markers may be more challenging and precludes the potential development of a large-scale DNA methylation analysis tool. Importantly, novel targeted approaches for epigenetic age prediction are being developed, including methods based on droplet digital PCR (ddPCR) [35].

In this study, we aimed to develop and compare the performance of three promising HTS enrichment protocols for targeted highly multiplexed DNA methylation analysis, including hybridization-based SureSelect^XT^ Methyl-Seq and Bisulfite Padlock Probes protocols followed by sequencing on MiSeq FGx and amplicon-based Ion AmpliSeq™ method integrated with Ion Torrent S5. Assays were designed using a predefined set of 161 CG/CA sites covering markers included in Hannum [2], Woźniak [33], Belsky [36] and Zhang [37] models developed to predict age and other age-related parameters in blood. The performance, scalability, and feasibility of the assays were assessed, and the obtained DNA methylation quantifications were compared between HTS methods and with genome-scale data generated by EPIC technology. Finally, methylation data for blood samples generated with the use of individual technologies were used to evaluate the performance of predictive models associated with the markers.

## Results

### Design of target methylation panels

Using in silico tools and/or company support, custom primer/probe panels were developed to analyze DNA methylation in predefined regions of the genome (Additional file 1: Table S1). Probes targeting 100% of the targets were successfully designed for both hybridization-based technologies, i.e. SureSelect and Bisulfite Padlock Probes (BSPP). However, for two targets (cg26758386 snoU13 and cg11674508 RP11231P20.2) probes with reduced specificity values were noted at the design stage. The SureSelect panel included 52,185 probes covering 120 genomic regions with 95 cytosines analyzed on the Watson and 66 cytosines on the Crick strands (Additional file 1: Table S1). The custom BSPP panel included 300 DNA probes targeting 124 genomic regions and 161 cytosines. The 92 cytosines in the panel were analyzed on the Watson strand and the remaining 69 cytosines on the Crick strand. The panels contained a higher number of probes than expected due to the degeneracy of the probes or to increase the chances of the probe binding to the target site in the genome (overlapping probes).

For the Ion AmpliSeq method, the panel design was successful at 96.3%. For 6 out of 161 cytosines, primer design failed despite attempts to relax the specificity requirements, due to the location of the cytosines in difficult, repetitive parts of the genome. For 134 cytosines, primers targeting both strands of DNA (W and C) were successfully designed; for 16 cytosines methylation was analyzed only on the Watson strand, and for the remaining 5 on the Crick strand. Ultimately, 1019 primers were designed for 216 amplicons (125–175 bp amplicon size), including primers for two lambda control amplicons. On average, 5–6 oligonucleotides were designed per amplicon again due to degeneracy caused by CpG sites present in primer binding sites.

### Read depth analysis

All sequencing runs yielded high total coverage. The theoretical capacity of a single flowcell used in the experiments was greater (MiSeq Reagent Kit v3; 25 M) than a single chip (Ion 530™ Chip; 15–20 M) but in both cases 4 barcoded libraries were processed together and sequenced. The mean mapped read depth per sample was 1,041.3  ± 371.7 k (median: 999.9 k; range: 563.2–1881.9 k) for Ion AmpliSeq, 237.5  ± 123.6 k (median: 227.6 k; range: 37.2–451.5 k) for SureSelect and 817.6  ± 580.8 k (median: 962.8 k; range: 78.1–1794.3 k) for BSPP sequencing runs, as measured across sequencing runs within a repeatability study. Although the mapping efficiency was high (85.6 ± 4.9%) with Ion AmpliSeq technology, the lower than expected number of mapped reads was due to the low rate of chip loading (in the range of 28–47%), which according to the manufacturer's protocol is expected for this application. In turn, the much lower than expected read depth for hybridization-based technologies was caused by the high level of off-target reads. Although the mapping efficiency was relatively high for the SureSelect (76.2 ± 6.9%) technology, only 7.3 ± 3.1% of the reads were mapped to the target. High levels of off-reads were found for probes targeting 4 regions (cg20822990 ATP13A2/SDHB, cg11674508 RP11231P20.2, cg25428494 HPSE, and cg26758386 snoU13), including two with low specificity marked at the panel design stage. For the BSPP protocol, the mapped reads were at the level of 37.2 ± 11.9%, and the on-target parameter ranged from 1 to 22% depending on the sequencing run.

**Fig. 1:**
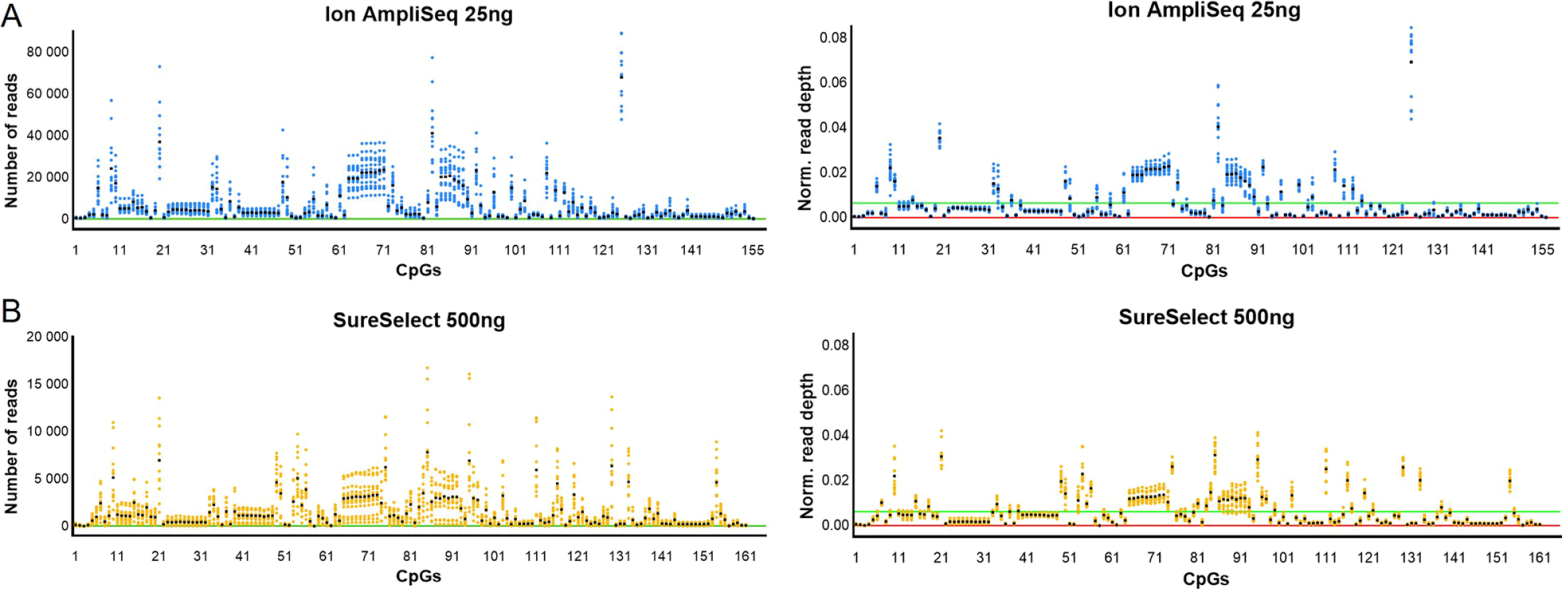
Distribution of reads across all cytosine targets and all sequencing runs performed in a repeatability study for Ion AmpliSeq (**A**) and SureSelect (**B**) technologies, using raw (left) and normalized (right) read depth. The green line on the raw read depth graphs indicates the minimum number of reads set to 50, while the green line on the normalized read depth graphs shows an expected depth of 0.00065 for Ion AmpliSeq and 0.00060 for SureSelect, assuming a perfect distribution of reads between targets. The X-axis shows the target cytosines in the order given in Additional file 1: Table S1

The mean observed coverage per marker was 6,717.8 ± 9,910.2 (median: 2,922.0; range: 32–89,111), 1,475.4 ± 1,972.7 (median: 717.5; range: 0–16,745) and 5,078.2 ± 24,918.0 (median: 79.0; range: 0–611,782) for Ion AmpliSeq, SureSelect, and BSPP, respectively. While the amplicon-based approach was less effective in the in silico design phase, it provided the greatest read coverage. Figure 1 shows raw and normalized read depth across all markers. In the Ion AmpliSeq protocol, 99.4% of targets were covered more than 200 × and 81.9% more than 1000x (Additional file 2: Table S2). For the SureSelect technology, 83.9% of cytosines were covered more than 200x and 47.2% more than 1000x. There was one CpG marker (cg09404119 MIR4456) with no coverage, and the other marker (cg11674508 RP11231P20.2) had less than 20 reads 78.6% of the time. For the best of the three sequencing runs performed for the BSPP technology, 42.9% of the targets were covered more than 200 × and only 14.3% of the targets more than 1000x. For 43 CpGs (26.7%) no reads were obtained and this number refers to experiments done after optimization, i.e. probe rebalancing. Unfortunately, despite the success initially achieved at the design stage of the BSPP probe panel, a very large percentage of probes failed, and the accuracy of DNA methylation quantification was significantly lower (Additional file 2: Fig. S1 and S2) than with the other two HTS methods used, and therefore, further experiments were abandoned at this stage for this technology. High bisulfite conversion was confirmed, determined to be 99.8 ± 0.1% and 98.3 ± 0.3% for Ion AmpliSeq and SureSelect, respectively.

### Sensitivity study

**Fig. 2:**
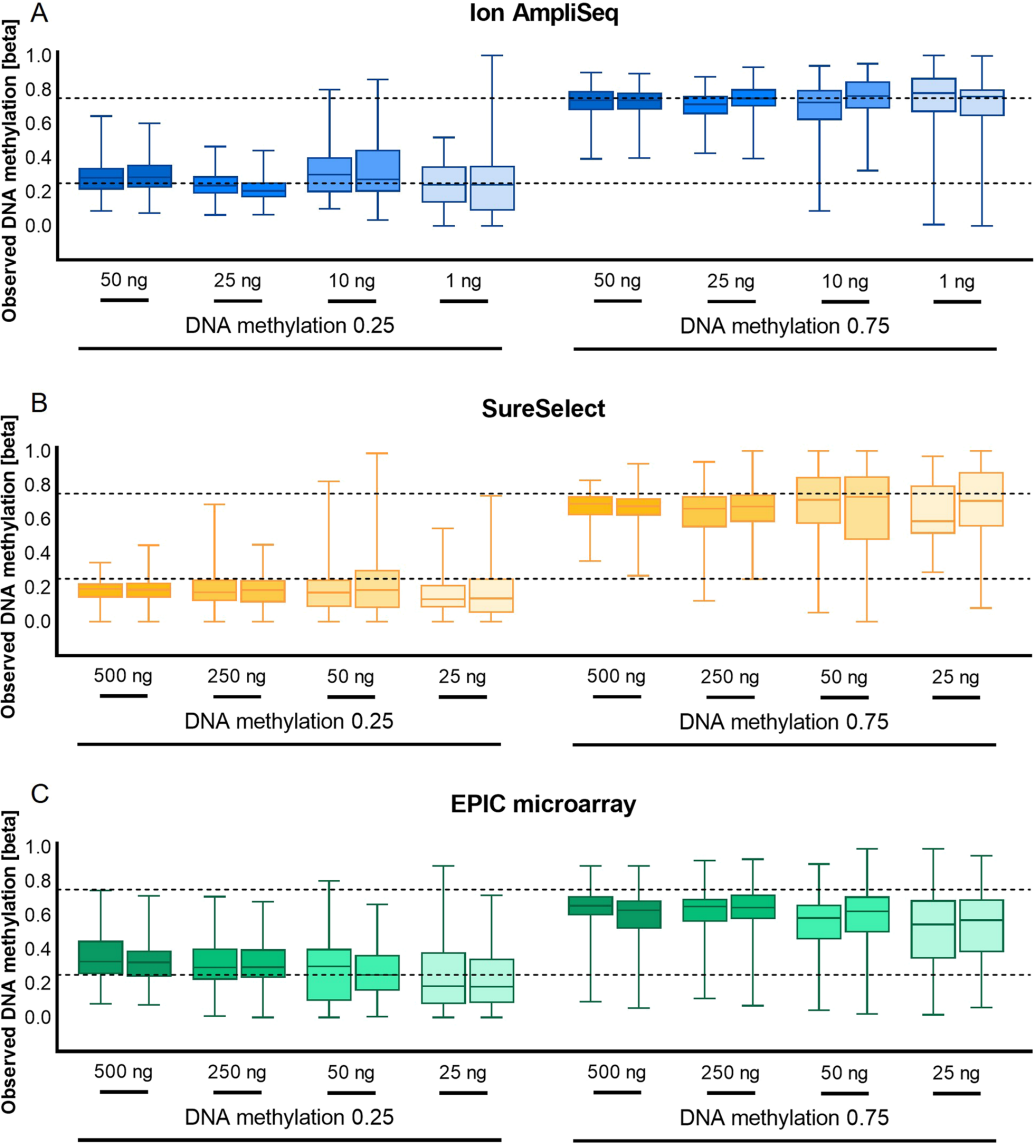
The results of the sensitivity study for Ion AmpliSeq (**A**), SureSelect (**B**), and EPIC (**C**) technologies conducted on DNA methylation standards, for two DNA methylation beta values (0.25 and 0.75) on low and high DNA inputs. DNA methylation measurements are shown across all studied cytosines. The dashed lines indicate the expected value of DNA methylation

For the sensitivity study, input DNA dilution series from 50 ng down to 1 ng for Ion AmpliSeq and from 500 ng down to 25 ng for SureSelect were tested for two DNA methylation beta values, 0.25 and 0.75. Although a different range of starting DNA was analyzed depending on the technology, two common points were used, 50 and 25 ng. We observed the lowest variability in DNA methylation quantification for 25 ng of DNA for Ion AmpliSeq and 500 ng of DNA for SureSelect (Fig. 2). For Ion AmpliSeq, the mean observed methylation beta value for the 0.25 and 0.75 methylation controls, measured across all targets and through both technical replicates, was 0.226 ± 0.068 and 0.722 ± 0.086, respectively. An increase in the standard deviation was observed for lower DNA inputs. For the SureSelect technology, the observed methylation for the highest DNA input (500 ng) was 0.185 ± 0.063 for the 0.25 DNA methylation standard and 0.669 ± 0.094 for the 0.75 methylation standard, and there was an increase in the standard deviation observed for smaller DNA inputs.

We also performed a sensitivity study for the EPIC microarray technology and observed similar variability in DNA methylation measurements for the two highest DNA inputs, 500 and 250 ng, with mean observed methylation values at 0.340 ± 0.127/0.620 ± 0.134 and 0.311 ± 0.130/0.632 ± 0.125, respectively (Fig. 2). An increase in result variability and standard deviation was observed for 50 ng and 25 ng of input DNA.

### Repeatability study

**Fig. 3:**
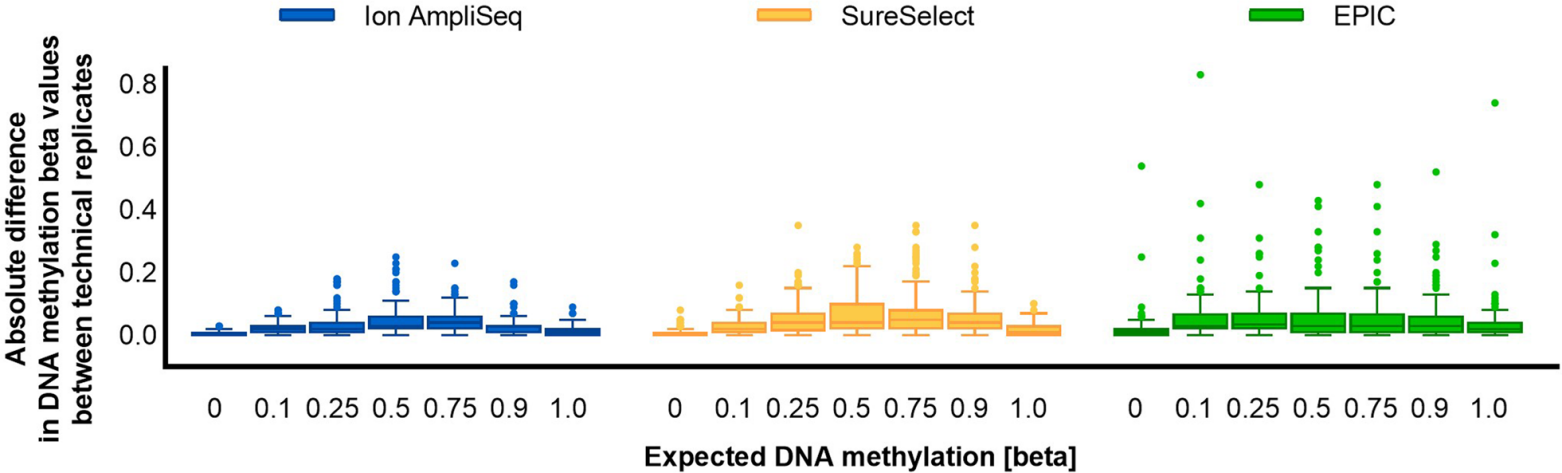
Boxplots showing the mean absolute difference between technical replicates, measured across all DNA methylation beta values and all cytosines

**Fig. 4:**
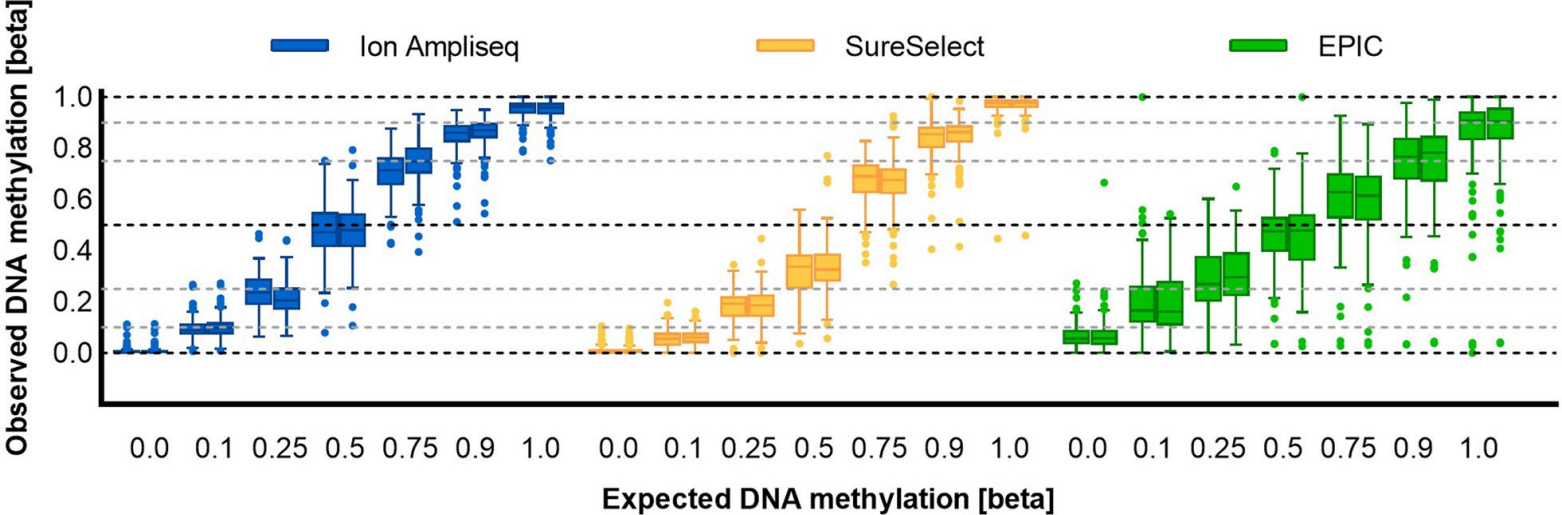
DNA methylation levels measured across all CG/CA targets in duplicate standard samples of seven selected beta values (0, 0.1, 0.25, 0.5, 0.75, 0.9, 1) to assess the reproducibility of the results

We noted a high level of correlation of DNA methylation measurements between replicates, with Spearman R equal to 0.993 for Ion AmpliSeq, 0.984 for SureSelect, and 0.956 for EPIC, as measured across all DNA methylation beta values. The lowest mean absolute difference between technical replicates was recorded for Ion AmpliSeq (0.027 ± 0.033) and was slightly higher for SureSelect and EPIC (0.041 ± 0.052 and 0.048 ± 0.075, respectively; Fig. 3). Plotting the observed DNA methylation levels, we observed the expected linear increase in methylation for successive DNA methylation beta values (Fig. 4). Overall, measured methylation values had mean standard deviations of 0.059 for Ion AmpliSeq, 0.060 for SureSelect, and 0.130 for EPIC, measured across all DNA methylation beta values. Interestingly, the variability of DNA methylation results depended on the DNA methylation beta value and was the highest for the intermediate methylation values and the smallest for the extreme ones, and this applies to all tested technologies (Figs. 3 and 4).

### Methods comparison

**Fig. 5:**
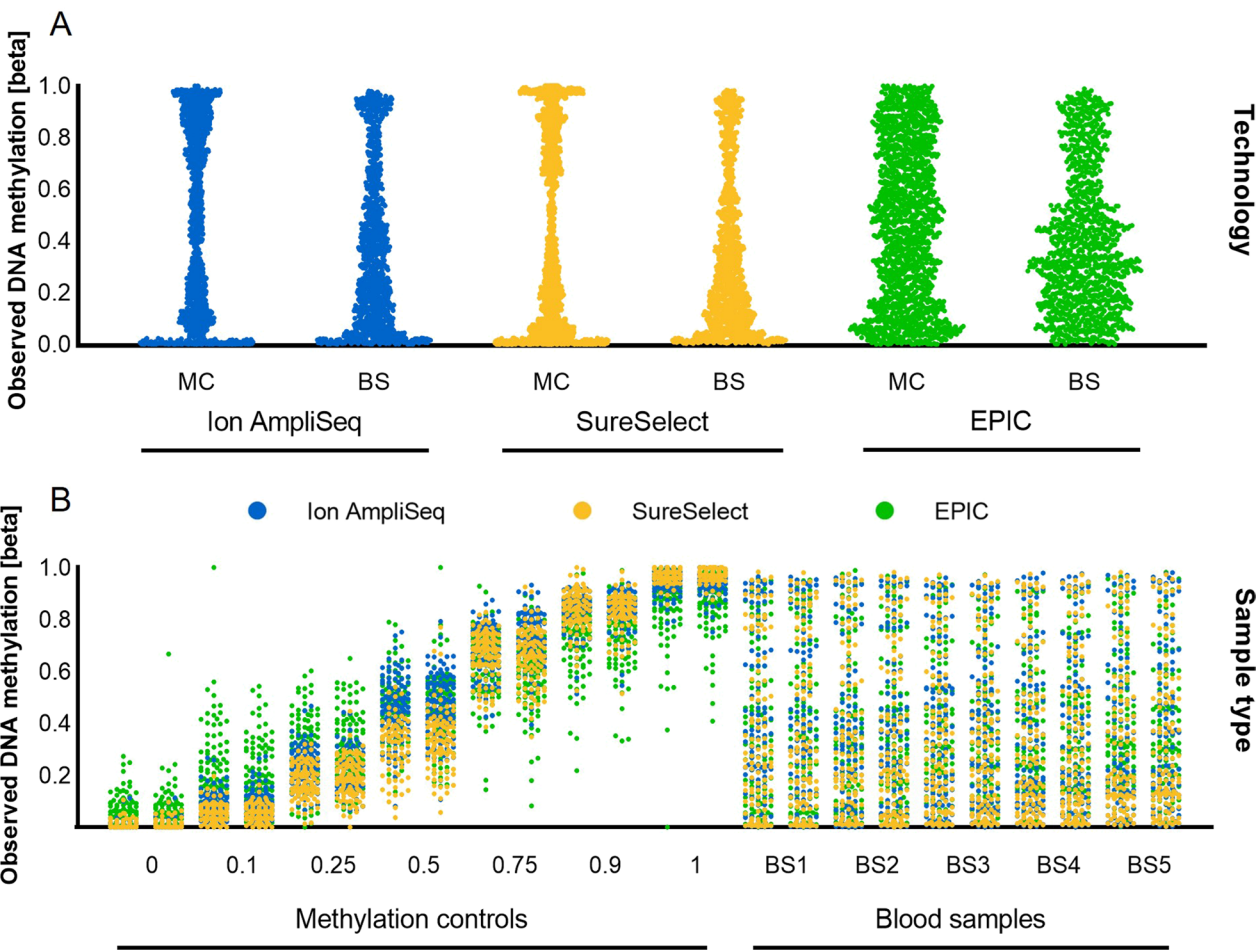
Distribution of DNA methylation measurements across 116 CG/CA targets shared between three technologies plotted by the assay type (**A**) or sample type (**B**). Each point corresponds to one measurement for one cytosine in one sample. Colors indicate assay technology. MC: methylation controls; BS: blood samples

**Fig. 6:**
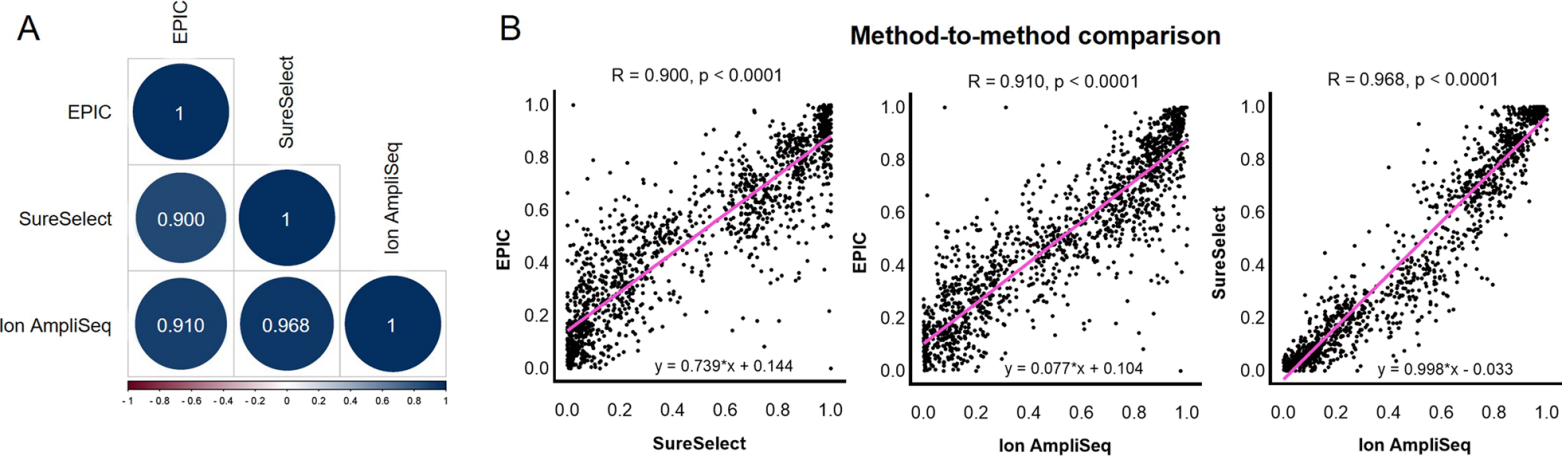
Comparison of the methods. Heatmap of the Spearman correlation matrix for cross-test comparisons across all DNA methylation measurements done within the repeatability study (**A**). Scatterplots illustrating cross-method correlations (**B**). Pink lines indicate fitted linear models, and the reported numbers (*R*) are Spearman correlation coefficients. More than 1550 comparisons were considered for 116 targets shared between all three technologies

The expected distribution of DNA methylation measurements was observed for all assays, both for DNA methylation controls and for blood samples (Fig. 5). However, HTS methods more frequently reported more extreme, highly or lowly methylated values when compared to EPIC microarray. The conducted analyses indicated good overall agreement in DNA methylation measurements between all technologies across 116 shared CpG sites (Fig. 6). The Spearman correlation equal to or above 0.9 was obtained for each pair of methods. Of both HTS methods, results more in line with EPIC were reported for the Ion AmpliSeq technology with a mean absolute difference of 0.106 ± 0.103.

**Fig. 7:**
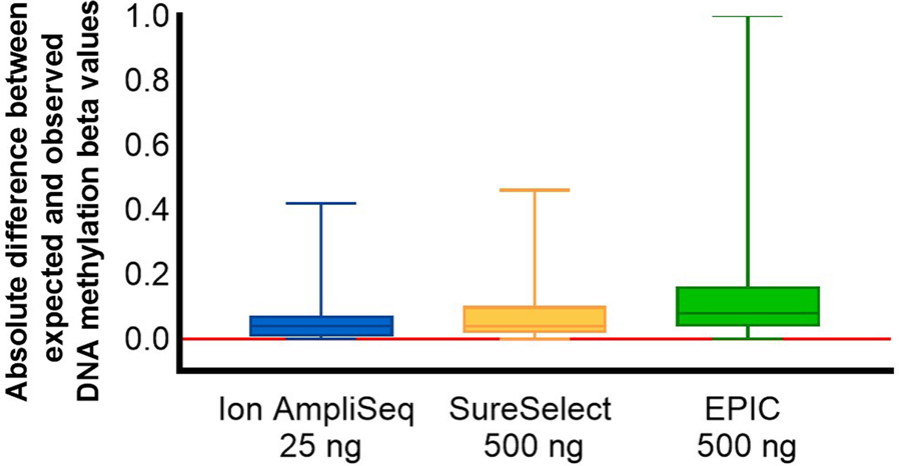
Boxplots summarizing the distribution of absolute differences between expected and observed DNA methylation measurements across 116 shared genomic targets

Comparing the accuracy of DNA methylation determination, the smallest mean absolute difference between the observed and expected DNA methylation values for methylation standards was obtained for the Ion AmpliSeq technology (0.054 ± 0.058), higher for SureSelect (0.070 ± 0.076) and the highest for EPIC (0.116 ± 0.107) (Fig. 7).

### Estimation of age-related parameters

**Fig. 8:**
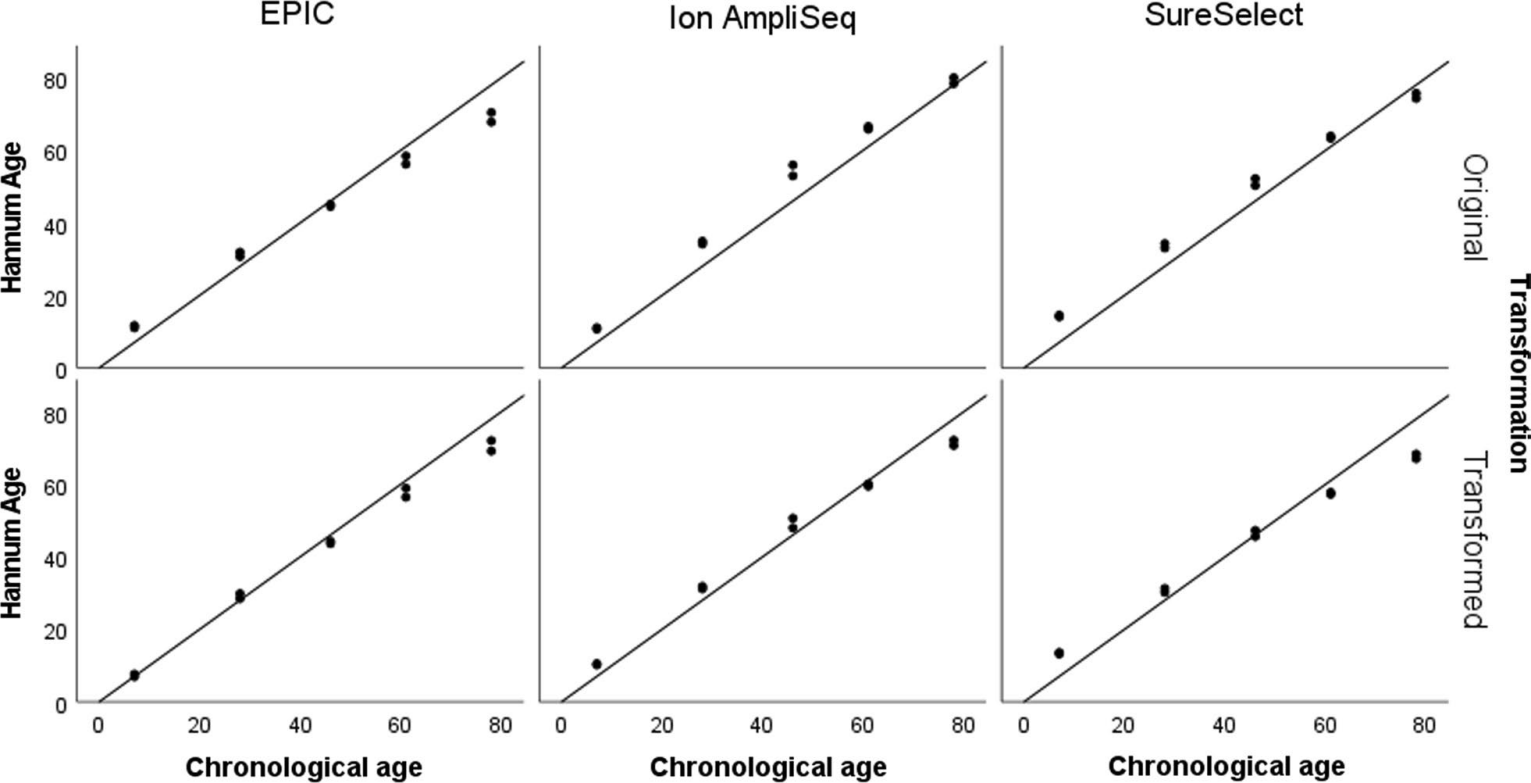
Data transformation by regressing prediction outputs on chronological age and its impact on age prediction accuracy using a Hannum clock applied on different types of data generated for five blood samples analyzed in duplicate. The reference line indicates the line of identity (y = x), i.e. the expected correlation of prediction results with chronological age. Data were transformed using following equations: HannumAge HTS Transformed = 0.64 + (HannumAge HTS*0.89); HannumAge EPIC Transformed = − 5.16 + (HannumAge EPIC*1.10)

To assess the performance of selected epigenetic clocks, we used DNA methylation data from 5 blood samples collected along with information about sex and age. Three of the four clocks were originally trained using data generated with microarray technology, the Hannum age clock, Zhang mortality risk score (MRS) and Belsky pace of aging (PoAm). The VISAGE blood age model was built using Illumina-based data. Of the 44 CpGs tested in VISAGE, only 13 are available on microarrays. Here, we used the available 13 CpGs to train the model using EPIC data generated for 249 blood samples collected as part of the Polish epigenome project (data not shown). Using stepwise regression, 6 CpGs were selected, with 5 loci overlapping between the two models (Table 1). The high accuracy of chronological age prediction was confirmed by the Hannum and VISAGE age models when applied to EPIC data with MAE = 3.9 and MAE = 2.9, respectively (Table 1). At the same time, applying the original Hannum age model to DNA methylation data generated by HTS technologies resulted in a higher prediction error. As shown in Fig. 8 and Additional file 2: Fig. S3–S6, the use of HTS data usually led to an overestimation of the prediction results when using models trained on EPIC. To overcome this issue, the linear transformation was applied, which increased the accuracy of Hannum age prediction for Ion AmpliSeq data to the level of MAE = 3.6 ± 2.0 (Fig. 8, Table 1). The transformation of the data also improved age prediction using the original VISAGE model applied on Ion AmpliSeq data (MAE dropped from 4.6 ± 2.6 to 2.7 ± 2.5, Table 1, Additional file 2: Fig. S3). Ion AmpliSeq and SureSelect data transformation has also been applied to the PoAm and Zhang MRS calculators (Additional file 2: Fig. S4–S6). Interestingly, in most cases, the use of data transformation also reduced the observed differences between technical replicates (Table 1).

**Table 1:** Summary statistics and comparison of age-related parameters collected for blood samples *N* = 10 with a mean age = 44.0 ± 26.1 (age range 7–78)

Epigenetic clock	Statistics	EPIC		Ion AmpliSeq		SureSelect	
		Original	Transformed	Original	Transformed	Original	Transformed
VISAGE blood age	Mean predicted value ± SD	40.3 ± 26.1^*^	NA	46.9 ± 29.6^**^	41.8 ± 27.4	42.6 ± 29.6^**^	NA
	Mean difference between replicates	2.9 ± 2.0	NA	1.2 ± 0.4	1.2 ± 0.4	2.5 ± 1.6	2.3 ± 1.5
	Mean absolute error of prediction (MAE)	3.9 ± 2.9	NA	4.6 ± 2.6	2.7 ± 2.5	3.9 ± 2.7	NA
Hannum age	Mean predicted value ± SD	42.7 ± 21.2	41.7 ± 23.3	49.0 ± 25.4	44.5 ± 22.7	47.5 ± 22.7	43.1 ± 20.3
	Mean difference between replicates	1.5 ± 0.9	1.6 ± 1.0	1.3 ± 1.0	1.2 ± 0.9	1.1 ± 0.6	1.0 ± 0.5
	Mean absolute error of prediction (MAE)	4.3 ± 2.8	2.9 ± 2.8	5.0 ± 2.7	3.6 ± 2.0	4.7 ± 1.9	4.8 ± 3.5
PoAm	Mean predicted value ± SD	1.02 ± 0.09	NA	1.11 ± 0.08	1.04 ± 0.06	1.11 ± 0.09	1.03 ± 0.06
	Mean difference between replicates	0.05 ± 0.04	NA	0.05 ± 0.04	0.03 ± 0.03	0.04 ± 0.02	0.03 ± 0.01
	Mean absolute error of prediction (MAE)	NA	NA	NA	NA	NA	NA
Zhang MRS	Mean predicted value ± SD	− 2.63 ± 0.42	NA	− 1.52 ± 0.60	− 2.64 ± 0.36	− 1.12 ± 0.52	− 2.40 ± 0.32
	Mean difference between replicates	0.11 ± 0.09	NA	0.15 ± 0.12	0.09 ± 0.07	0.08 ± 0.08	0.05 ± 0.05
	Mean absolute error of prediction (MAE)	NA	NA	NA	NA	NA	NA

The results include the original outputs of the models and the results after applying a linear data transformation that allowed correction for the observed technological differences (EPIC vs. HTS)

^*^A new version of the VISAGE model was developed using alternative list of CpGs (ELOVL2 cg16867657 chr6:11,044,644, KLF14 cg08097417 chr7:130,734,372, MIR29B2CHG cg10501210 chr1:207,823,675, FHL2 cg22454769 chr2:105,399,310, PDE4C cg17861230 chr19:18,233,091 and EDARADD cg09809672 chr1:236,394,382) and EPIC data for training

^**^Original VISAGE age model developed by Woźniak et al. was used trained on 6 CpGs (ELOVL2 chr6:11,044,634, KLF14 chr7:130,734,375, MIR29B2CHG chr1:207,823,681, FHL2 cg06639320 chr2:105,399,282, TRIM59 chr3:160,450,202, PDE4C chr19: 18,233,127) and Illumina sequencing data

The final prediction results were subjected to correlation analysis, yielding a high level of correlation (*R* = 0.99) between Hannum's age and VISAGE (Additional file 2: Table S3). Zhang MRS achieved a correlation of *R* > 0.7 with Hannum age and VISAGE, while PoAm was not significantly correlated with any of the other clocks nor with chronological age, but small sample size should be considered (*N* = 10) (Additional file 2: Fig. S7–S8). The obtained result suggests the independence of the information coming from the PoAm and Zhang MRS models from the information contained in the VISAGE and Hannum models, which may be clinically significant. Importantly, we observed a high correlation of the results obtained for individual clocks using different technologies used to collect methylation data (*R* > 0.9 for all clocks except PoAm; for the PoAm calculator, a correlation of 0.78 was observed for the PoAm values obtained with EPIC vs. Ion AmpliSeq; Additional file 2: Table S4).

## Discussion

DNA methylation analysis is gaining increasing interest in biomedical and forensic research. To increase the use of DNA methylation analysis in laboratories performing routine diagnostics, it is necessary to adapt standard genotyping methods and protocols to the analysis of bisulfite-converted DNA. Here, we demonstrated robust quantification of DNA methylation values using two customized HTS-based protocols, Ion AmpliSeq, and SureSelect. The assays interrogate 161 CG/CA sites in the genome targeted by multiplex PCR or probes hybridization protocol combined with Ion Torrent S5 vs. MiSeq FGx sequencing. The protocols were characterized by high accuracy of DNA methylation quantification, and we have demonstrated the high utility of the developed laboratory protocols for the determination of parameters related to epigenetic aging with MAE of chronological age prediction below 5 years for Hannum and VISAGE clocks.

All three HTS-based protocols met the basic criterion of panel customization and multiplexing. Agilent SureSelect^XT^ Methyl-Seq is an RNA probe-based technology [38, 39]. What sets this technology apart from other protocols is the type of target enrichment that is performed on fragmented genomic DNA before bisulfite conversion occurs. This offers great potential for multiplexing methylation markers but at the cost of increased input of genetic material. Although the SureSelect method provided good accuracy of DNA methylation measurements in general, some weaknesses should be noted, including high DNA input, relatively long library preparation protocol with DNA fragmentation step requiring initial optimization, and relatively high cost. The second protocol chosen also used probe hybridization for target enrichment, but the probes differ significantly in structure. The BSPP method is a library-free and non-commercial approach originally developed for genome-scale analysis of DNA methylation [40, 41]. It seems that the limiting step in BSPP technology is the time-consuming step of balancing individual probe concentrations and optimizing reaction conditions [55]. For the amplicon-based method used, six cytosines were missing from the panel due to difficulties in designing primers with sufficient specificity. On the other hand, it was noticed that the targets missing in the amplicon-based technology partially coincide with the problematic targets in SureSelect technology.

Although the hybridization protocols have greater multiplexing capabilities, the best all-round performance was observed in our study for amplicon-based protocol. Ion AmpliSeq is a well-established technology that enables efficient and scalable analysis of genetic variability in up to thousands of target genomic regions, using a small amount of starting DNA, e.g. [42, 43]. Thermo Fisher Scientific recently adopted Ion AmpliSeq technology for measuring DNA methylation by launching a community panel for cancer research [44, 45]. The utility of Ion AmpliSeq technology for targeted analysis of DNA methylation was further corroborated by another group [46]. Importantly, the amplicon-based technology applied in Ion AmpliSeq protocol uses a strategy of analyzing CpG markers from both strands of DNA. Other advancements of Ion AmpliSeq over probe-based protocols are ease of protocol, reduced sample-preparation time and a user-friendly bioinformatics pipeline integrated with the Ion Torrent Server which reduces the list of external programs needed to perform data analysis. Reduced chip loading is the main limitation of the Ion AmpliSeq technology, increasing the final cost of analyses.

Forensic genetics is particularly demanding in terms of the sensitivity of the methods used [16]. We have demonstrated that the Ion AmpliSeq assay can process smaller amounts of input DNA compared to other methods, providing a robust quantification of DNA methylation values down to 25 ng of DNA. Although the Ion AmpliSeq technology was able to process even smaller amounts of DNA, an increase in the variability of results was observed for 10 ng and 1 ng DNA, which is a known phenomenon due to the quantitative nature of DNA methylation and the stochastic effects that occur when the number of DNA molecules is limited [17]. A similar threshold of 20 ng of DNA needed to precisely measure DNA methylation across 8 loci was determined for the VISAGE models [32–34].

In 2016, Bock et al. conducted a large-scale multicenter validation study of various available DNA methylation analysis methods, both epigenome-wide and targeted [44]. The authors showed a good consistency of results across all the methods used, but at the same time, they pointed to the advantage of amplicon bisulfite sequencing and pyrosequencing [44]. A systematic comparison of DNA methylation data collected by different technologies was also conducted by Freire-Aradas [18]. Bisulfite amplicon sequencing offers outstanding quality in terms of sensitivity, accuracy, analysis cost, and throughput [19, 29]. On the other hand, it is suggested that whole-genome bisulfite sequencing may be superior to PCR-based methods, providing the most reproducible and accurate measurements of DNA methylation and avoiding amplification bias issues, but the cost of WGBS and bioinformatics demands remain much higher [47].

The protocols developed in this study cover methylation markers of four epigenetic clocks established in recent years and represent various parameters related to aging processes. Importantly, we showed that data transformation can eliminate differences between technologies and enables the application of models trained with microarray data on HTS sequencing data. We observed a high method-to-method correlation between DNA methylation measurements, but at the same time, we observed a systematic difference of about 10% on average between HTS and EPIC technologies and about 6% between both HTS technologies. By collecting data using different technologies for the same set of samples, we determined the data shift pattern and made appropriate adjustments. Z-score data transformation has also been applied in previous studies regarding the application of age prediction models to methylation data generated by different technologies and even different types of the same apparatus [18, 23, 48].

As age-related changes in the body increase the risk of various diseases and disabilities [49], a comprehensive approach to treatment based on slowing down the aging process is now being suggested [36, 50]. Measurements of the rate of aging can be useful in disease risk assessment and longitudinal studies to track the body's response and changes in aging to given drugs, diet, or specific lifestyle recommendations. In forensic science, the estimation of a person's chronological age based on the analysis of biological traces can be an invaluable tool at the investigation stage [22, 25]. We accurately predicted the chronological age in the data generated by each data collection technology using two age estimators, VISAGE and the Hannum clock. The VISAGE tool was developed to provide a compact set of markers for rapid and accurate age prediction in various somatic tissues for forensic purposes [33]. Hannum's clock, on the other hand, contains a broader set of CpGs (71), and although a high level of correlation between chronological age and epigenetic age is observed for this clock, a result that deviates from a person's chronological age may have biological significance [2]. The Zhang et al. and PoAm estimators represent the next generation of epigenetic clocks, and the prediction result is not provided here in units of years. Zhang et al. model uses 10 CpGs to estimate a mortality risk score on a continuous or categorical scale that has been reported to be strongly associated with all-cause mortality [37]. A mortality risk score of 1, 2–5, > 5 means a two-, three- and seven-fold increased risk of death compared to a score of 0, respectively. Belsky et al. calculator returns the result in z-score units, which can be interpreted as the number of years of physiological decline per one calendar year. Importantly, people with elevated PoAm levels were also perceived as looking older, which can be very useful in forensics when creating a genetic sketch of the offender [14]. On the other hand, it has been shown that people with a lower PoAm level have generally better life parameters, greater physical fitness and greater mental activity [36, 51].

Interestingly, PoAm was reported not to correlate well or correlate only moderately with other epigenetic clocks [36, 52], which is consistent with the results obtained in the present study. Aging results from the accumulation of different changes at the cellular level, and importantly, it is suggested that different measures of biological aging may not necessarily measure the same aspect of aging [51, 52]. It, therefore, seems that the combination of models used in this project represents a good cross-section of available tools for the assessment of epigenetic aging and can provide relatively broad information while maintaining a relatively reasonable number of markers.

While the laboratory protocols developed here require further validation, particularly for forensic applications where difficult samples are to be dealt with, they can be a good starting point for developing a practical tool for geroscience, diagnostics, or criminalistics. Importantly, the Ion AmpliSeq technology used in this study is characterized by high flexibility, enabling easy expansion or modification of the existing panel, which opens up new possibilities for the development of DNA methylation applications in practice in the near future. We have demonstrated the superiority of HTS assays over microarray technology in terms of the accuracy of methylation measurement, particularly with respect to low and high methylation values. The effect of EPIC under-methylation for high methylation levels and over-methylation for low methylation levels has been described in the literature. Therefore, the use of HTS technology may be useful for a broader list of epigenetic estimators, including mitotic clocks that use unmethylated CpGs in fetal tissue, and the flexibility of the HTS technologies provides opportunities for further exploration in this area. While the HTS technology for DNA methylation target analysis has its advantages, it also has its limitations. The proposed tool allows to obtain information on epigenetic aging only in the range of four clocks, while new models are constantly being developed, taking into account the analysis of hundreds of markers [53–56]. Microarray technology offers comprehensive possibilities in this area. It is still the gold standard in methylation research, allows easy comparison of results between models, provides access to large data sets collected over the years, and due to the high usability of the method, it continues to be updated [57]. Furthermore, all epigenetic calculators used in our project, in accordance with the original studies, were developed without the need to apply the correction for blood cell type composition. However, the inclusion of markers to assess cellular composition would significantly broaden the relevance and application of the developed tool and should be considered in future.

## Conclusions

This study yielded several important conclusions and discoveries. We confirmed previous reports showing that high-throughput sequencing methods, known for the efficient analysis of genetic polymorphisms, are also suitable for the analysis of DNA methylation. The two methods selected for detailed evaluation, i.e. Ion AmpliSeq and SureSelect followed by Ion Torrent S5 and MiSeq FGx sequencing, respectively, enabled large-scale multiplexing and provided precise and repeatable measurements of DNA methylation and enabled accurate estimation of epigenetic aging-related parameters. The protocols showed robust quantification of DNA methylation with a mean absolute difference in methylation beta value between replicates below 0.05 and a mean absolute difference between expected and observed methylation beta values ≤ 0.07. In addition, we show that with the use of data transformation, models originally trained on microarray data can be successfully applied to sequencing data. The Ion AmpliSeq method can be particularly recommended for routine use in DNA laboratories due to its flexibility of panel design, user-friendly lab protocol, high accuracy, low variability down to 25 ng, streamlined data analysis, and associated high precision of age estimation. The developed panel allows accurate and sensitive analysis of 161 CpG sites which are compatible with four predictive models for age and age-related features and can be useful in forensic, medical and healthcare applications.

## Methods

### Preparation of DNA samples

Experiments were performed using artificially methylated standards and blood samples. Fully methylated (100%) and unmethylated (0%) controls from the human WGA methylated & non-methylated DNA Set (Zymo Research, Irvine, California, USA) were mixed in appropriate proportions to obtain the desired values of DNA methylation (0, 0.10, 0.25, 0.50, 0.75, 0.90, and 1). To assess the performance of epigenetic clocks covered by the analyzed markers, 5 blood samples from unrelated individuals were also collected, ensuring an adequate representation of the subjects’ age (7, 28, 46, 61, and 78 years). Samples were gathered from the volunteers as part of a larger cohort representing the general population of Poland. The study was approved by the Bioethics Committee of the Jagiellonian University in Krakow (decision no. 1072.6120.132.2018) and the participants provided written informed consent.

Blood samples were DNA extracted by an automated method and the Maxwell RSC Blood DNA Kit (Promega Corporation, Madison, USA). Subsequently, all samples were quantified using the Qubit dsDNA HS Assay Kit, evaluated for quality using the NanoDrop 2000 Spectrophotometer (Thermo Fisher Scientific—TFS, Waltham, MA, USA), and normalized to concentrations appropriate for the experiment.

### HTS assays selection

A literature review was conducted to select the most promising targeted high-throughput sequencing protocols for DNA methylation analysis. The basic criterion for the selection of methods was panel customization and the ease and scale of multiplexing. As a result, three protocols were pre-selected. Two of them, SureSelect^XT^ Methyl-Seq (SureSelect; Agilent Technologies, Santa Clara, CA, USA) and Bisulfite Padlock Probes (BSPP, [41]), use probe hybridization technique for target enrichment and are followed by sequencing on MiSeq FGx, while the third technology, which combines bisulfite protocol with the Ion AmpliSeq™ Library Kit Plus is an amplicon-based method integrated with Ion Torrent S5 sequencing (Ion AmpliSeq; Thermo Fisher Scientific). Selected protocols were systematically compared for determining DNA methylation in sensitivity and repeatability studies.

### Selection of target CpG sites and models

Our assays were designed to target 161 CG/CA genomic sites across four compact models selected to represent and predict various parameters of aging. The Hannum clock includes analysis of 71 CpG sites in the genome and predicts chronological age in blood with *r* = 0.91 and MAE = 4.9 years [2]. VISAGE models were trained using a precisely selected, compact list of 8 loci (44 CpGs) to accurately predict chronological age for forensic purposes. High accuracy of prediction was reported for blood (6 CpGs; MAE = 3.2), buccal swabs (5 CpGs; MAE = 3.7), and bones (6 CpGs; MAE = 3.4) [33]. The model of Zhang et al. estimates the mortality risk score (MRS) on a continuous or categorical scale of 1 to 10 based on the analysis of only 10 CpG sites in the genome [37]. The model presented by Belsky et al. allows the estimation of the pace of aging parameter (PoAm), which reflects the physiological change per one calendar age, based on the analysis of 46 CpG markers [36]. There are nine CpGs overlapping between the VISAGE and Hannum clock panels and one CpG common to the MRS and PoAm estimators. The list of cytosines is provided in Additional file 1: Table S1.

### Experimental design and assay performance

For the assay’s performance assessment, methods’ sensitivity, repeatability, and accuracy of DNA methylation assignment were tested.

(i) For sensitivity evaluation, libraries for 16 DNA methylation control samples per technology were prepared and sequenced. These included control samples prepared for 4 different DNA inputs in duplicates at two selected DNA methylation beta values (0.25 and 0.75). The range of DNA inputs tested varied by technology and was adjusted to cover optimal amounts of DNA recommended by the manufacturer's protocol.
(ii) For the study of reproducibility, 14 libraries were prepared for seven control samples at specific DNA methylation beta values (0, 0.1, 0.25, 0.5, 0.75, 0.9, and 1) analyzed in duplicate with a single DNA input, selected based on the results obtained in the sensitivity test (25 ng for Ion AmpliSeq and 500 ng for SureSelect).
(iii) The results of the sensitivity and reproducibility studies allowed us to assess the accuracy of the DNA methylation assignment and compliance with expected values, given the specified DNA input values and the minimum number of reads. Read depth and uniformity of amplicon coverage were also investigated.
(iv) To assess the performance of selected epigenetic clocks, 10 additional libraries were prepared for 5 blood samples from individuals of known age and sex, analyzed in duplicate for the optimal amount of input DNA.

The final number of libraries sequenced was 36. Four samples were processed together and sequenced per one chip or flowcell, resulting in a total of 9 sequencing runs per technology. Replicates were always analyzed on the same flowcell or chip. The level of DNA methylation for individual cytosines was presented in the form of beta values and ranged from 0 (completely unmethylated) to 1 (fully methylated). Beta value is calculated as the ratio of methylated (C or G) reads to the sum of methylated and unmethylated reads (C + T or G + A). It is assumed that the methylation values determined in this way correspond to the beta methylation values determined in the EPIC technology [58]. Importantly, all statistics provided for HTS technologies used an experimentally determined threshold of a minimum of 50 reads. The analyses performed showed only small differences in the precision of DNA methylation determination and overall variability of results using reads thresholds of 1000, 200, and 50 (Additional file 2: Fig. S9). Therefore, for results with a read depth of less than 50, missing data were considered. Statistical comparisons were performed with Microsoft Excel and IBM SPSS Statistics 28.0.1.0. or *R* (https://www.r-project.org/). [59].

## Panel design, library preparation, sequencing protocol, and HTS data analysis

### Ion AmpliSeq™ targeted sequencing technology

The custom Ion AmpliSeq primer panel was designed in silico with support from Thermo Fisher Scientific. The bisulfite conversion of DNA samples was performed using the MethylCode Bisulfite Conversion Kit (Thermo Fisher Scientific). The amount of DNA determined for a given experiment (50, 25, 10, or 1 ng) in an initial volume of 20 µl was converted and eluted in 10 µl. All 10 µl of bisulfite-treated DNA was used for library preparation according to the instructions provided in the “Bisulfite methylation library production and analysis using the Ion AmpliSeq™ Library Kit Plus” protocol. Targets were amplified using 5X Ion AmpliSeq™ HiFi Mix and a custom Ion AmpliSeq 5X primer pool with 25 cycles of PCR. At the test optimization stage, two rounds of primer rebalancing were performed and 38 lower-performing amplicons were spiked-in. After partial digestion of amplicons with FuPa reagent, the amplicons were ligated to the IonCode Barcode Adapters, purified with AMPure XP, and eluted in a master mix for library amplification. After 9 cycles of post-amplification, libraries were purified with the AMPure XP beads (Beckman Coulter) using 1.0X beads-to-sample volume ratio. Next, libraries were size-selected by a two-round purification with 0.5X beads-to-sample volume ratio of the AMPure XP beads. The barcoded libraries were evaluated using the Ion Library TaqMan Quantification Kit (QuantStudio 12 K Flex system, Applied Biosystems) and the High Sensitivity DNA Kit (2100 Bioanalyzer instrument, Agilent). DNA libraries for four samples were combined in equal ratios, normalized to 40–45 pM, templated, and sequenced using the Ion 520™ & Ion 530™ ExT Kit and Ion 530™ Chip on the Ion Chef™ Instrument and the Ion S5™ Sequencer, respectively (Thermo Fisher Scientific).

Sequencing results were reviewed and analyzed using Ion Torrent Suit Server 5.10.1. The methylation_analysis plugin was used to align the reads to the bisulfite-converted genome (GRCh38_Lambda), which was done using a modified version of the Bismark program. Then, methylated (ME) and unmethylated (UM) reads were counted and beta methylation values for target cytosines were derived, both on Watson (W) and Crick (C) strands. The final methylation call was made after summing the methylated and unmethylated reads from the amplicons from both strands, if available. The bisulfite conversion rate was evaluated using unmethylated Lambda DNA (Promega) added to the DNA sample prior to conversion according to the manufacturer's instructions.

### SureSelect^XT^ methyl-seq target enrichment system

The RNA probe panel targeting 161 cytosines was designed using SureDesign software and support from Agilent Technologies. Max Performance XT HS/ XT HS2/ XT LI/ QXT option was used to improve the capture of genomic targets and boost hybridization. After measuring the DNA concentration, the amount of DNA determined for the experiment (500, 250, 50, or 25 ng) in a final volume of 50 µl was fragmented using a Bioruptor® Pico sonication system (Diagenode) to obtain fragments of 100–175 bp. The fragmented DNA was used for HTS library preparation according to the SureSelect^XT^ Methyl-Seq Target Enrichment protocol for Illumina Multiplexed Sequencing (for 1 µg) with modifications dedicated to lower DNA inputs as presented in the “Agilent SureSelect^XT^ Methyl-Seq Applications with Low-Input DNA and Smaller Capture Libraries” protocol. Probes were hybridized overnight at 65 °C for 18 h. DNA libraries were converted using the EZ DNA Methylation Direct Kit (Zymo Research). 18 µl of the bisulfite-converted library was amplified by adjusting the number of cycles to the DNA input. All recommended fragment size assessments were done using the High Sensitivity DNA Kit. Libraries were then prepared for sequencing using a library concentration of 10–14 pM and 10% PhiX spike-in. Four samples were sequenced per one flowcell of MiSeq Reagent Kit v3 (2 × 150 bp) using the MiSeq FGx System.

Raw sequencing reads in fastq files were quality-checked with FastQC software, and adapters were removed with Trimmomatic 0.39. The trimmed reads were then aligned against in silico bisulfite-converted GRCh38 human genome reference using the Bismark 0.19.0 software [60]. Bam files were then sorted and indexed using Samtools [61] and reviewed with Integrative Genomics Viewer (IGV) [62]. The depth of coverage in the target regions was estimated using GenomeAnalysisTK-3.6 (GATK) [63]. Finally, the total number of reads per cytosine analyzed was counted using bam-readcount with the minimum mapping quality set to 30. Bisulfite conversion efficiency was evaluated by analyzing non CpG-Cs observed in a sample within the targets.

### Bisulfite padlock probes protocol

The custom BSPP panel was designed in silico using ppDesigner [41] and 300 DNA probes were synthesized as phosphorylated 98-nt oligonucleotides targeting 124 genomic regions and 161 cytosines. Further details of the experiments performed with bisulfite padlock probes, including the library preparation protocol, are provided in the Additional file 2.

### Epigenome-wide data collection

We used the Illumina Infinium Methylation EPIC microarray (Illumina, San Diego, CA, USA) to measure the total DNA methylation content of the sample. DNA sample degradation was assessed by 0.7% agarose gel electrophoresis and concentration measured using the Qubit dsDNA HS Assay Kit. To minimize the batch effect, DNA samples were randomized using the RANDOMIZE web-based application [64]. DNA methylation control samples and blood samples analyzed in duplicates in the total number of 36, identical to the samples used in the HTS experiments, were subjected to microarray analyses. Randomized samples were provided in 96-well plates for bisulfite conversion and microarray analysis to the external company Human Genotyping Facility (HuGe-F) Erasmus MC University Medical Center Rotterdam.

Primary quality control of the generated DNA methylation data was assessed by uploading raw idat files into the GenomeStudio software (Methylation Module v1.8) [65]. The Illumina internal controls and background subtraction were applied to the samples. The control metrics were generated based on the Illumina guide and the detection P-value greater than 0.05 was used for filtering poor-quality samples. For EPIC data analysis, the manifest file version v-1–0-b5, consisting of 865,918 probes was used. Methylation array analysis was done using *R* version 4.1.3.1. The preprocessIllumina() function from minfi Package was used for background correction and control normalization [66, 67]. Of the 161 CpGs selected in this study, only 124 were found to be covered by EPIC. The methylation level of the 124 shared probes was then extracted as beta values (0–1) and compared to methylation quantifications obtained by HTS methods.

### Aging-related parameters estimation

Individual DNA methylation aging parameters were generated for blood samples based on HTS- or EPIC-determined DNA methylation values and using mathematical models available in the form of R scripts (methylCIPHER *R* package used for Hannum age and Zhang MRS score; DunedinPoAm38 R package used for PoAm estimation) [68] or *β* parameters of the linear regression equations (VISAGE models). There are five markers from the Belsky model and one from the Hannum clock missing in the Ion AmpliSeq panel (Additional file 1: Table S1) because of the issues with primer design. On the other hand, out of the 10 CpG markers covered by the Zhang et al. model, 2 of them are not analyzed in the EPIC technology. Missing methylation beta values were imputed before applying epigenetic age clocks using a mean imputation method replacing missing values with the overall mean obtained from the Horvath online calculator webpage https://dnamage.genetics.ucla.edu/.

Since the Hannum, Zhang MRS, and PoAm models were trained on data generated using microarray technology, it was necessary to apply transformation of the prediction outputs to properly interpret HTS-based methylation data. Mathematical equations for transformation were derived using linear regression analysis of age prediction results obtained with both types of methylation data collection methods, i.e. microarrays and HTS (for PoAm and Zhang MRS scores) or regressing age prediction results on chronological age (for VISAGE and Hannum models), conducted on the data generated for an extended group of 76 blood samples collected as a part of the Polish epigenome project (data not present).

### Supplementary Information

**Additional file 1. Table S1** The list and characteristics of the studied cytosines.

**Additional file 2. Fig. S1** Raw reads number distribution (**A**) and normalized read depth (**B**) for three sequencing runs with three approaches to rebalance the probes performed for the BSPP technology. **Fig. S2** Accuracy of DNA methylation measurement for the BSPP technology. Libraries were prepared for 0.5 DNA methylation standards. **Fig. S3** Data transformation and impact on the accuracy of age prediction using the original VISAGE blood age model trained on Illumina sequencing data as applied on DNA methylation data generated with Ion AmpliSeq technology. Data were transformed using the following equation: VISAGE blood age Ion AmpliSeq TRANSFORMED = − 1.61 + (VISAGE blood age Ion AmpliSeq*0.93). **Fig. S4** Data transformation and impact on the accuracy of PoAm parameter estimation as applied on DNA methylation data generated with Ion AmpliSeq and SureSelect technology. Data were transformed using the following equation: PoAm HTS Transformed = 0.23 + (PoAm HTS*0.72). **Fig. S5** Data transformation and impact on the accuracy of Zhang categorical MRS parameter estimation as applied on DNA methylation data generated with Ion AmpliSeq and SureSelect technology. Data were transformed using the following equation: MRS Cat. HTS Transformed = 25.76 + 13.29*MRS Cont. HTS Transformed + 1.81*MRS Cont. HTS Transformed*MRS Cont. HTS Transformed. **Fig. S6** Data transformation and impact on the accuracy of Zhang continous MRS parameter estimation as applied on DNA methylation data generated with Ion AmpliSeq and SureSelect technology. Data were transformed using the following equation: MRS Cont. HTS Transformed = − 1.72 + 0.61*MRS Cont. HTS. **Fig. S7** Scatterplot of PoAm and chronological age correlation (*R*=0.024) after applying data transformation. **Fig. S8** Scatterplot of the correlation between MRS and chronological age (*R*=0.783) after data transformation, taking into account the continuous (A) and categorical (B) character of the MRS parameter. **Fig. S9** Analysis of the impact of the applied threshold of the minimum number of reads on the precision of DNA methylation determination: mean absolute difference between the observed and expected DNA methylation beta values and the standard deviation of the results. **Table S2** Cytosines reaching the requested read depth threshold for the technologies tested. **Table S3** Pearson correlation analysis for different age-related parameters and chronological age, using data generated with Ion AmpliSeq technology. **Table S4** Pearson correlation analysis of results obtained for individual clocks with different DNA methylation data collection technologies.

## Data Availability

The datasets used and/or analyzed during the current study are available from the corresponding author on reasonable request.
