# Supplementary material for: Introduction of a multiplex amplicon sequencing assay to quantify DNA methylation in target cytosine markers underlying four selected epigenetic clocks
Source: Clin Epigenetics. 2023 Aug 10;15:128. doi: 10.1186/s13148-023-01545-2 (PMC10416531; doi:10.1186/s13148-023-01545-2)
Supplement: Supplementary file 2 — Additional file 2. Fig. S1 Raw reads number distribution (A) and normalized read depth (B) for three sequencing runs with three approaches to rebalance the probes performed for the BSPP technology. Fig. S2 Accuracy of DNA methylation measurement for the BSPP technology. Libraries were prepared for 0.5 DNA methylation standards. Fig. S3 Data transformation and impact on the accuracy of age prediction using the original VISAGE blood age model trained on Illumina sequencing data as applied on DNA methylation data generated with Ion AmpliSeq technology. Data were transformed using the following equation: VISAGE blood age Ion AmpliSeq TRANSFORMED = − 1.61 + (VISAGE blood age Ion AmpliSeq*0.93). Fig. S4 Data transformation and impact on the accuracy of PoAm parameter estimation as applied on DNA methylation data generated with Ion AmpliSeq and SureSelect technology. Data were transformed using the following equation: PoAm HTS Transformed = 0.23 + (PoAm HTS*0.72). Fig. S5 Data transformation and impact on the accuracy of Zhang categorical MRS parameter estimation as applied on DNA methylation data generated with Ion AmpliSeq and SureSelect technology. Data were transformed using the following equation: MRS Cat. HTS Transformed = 25.76 + 13.29*MRS Cont. HTS Transformed + 1.81*MRS Cont. HTS Transformed*MRS Cont. HTS Transformed. Fig. S6 Data transformation and impact on the accuracy of Zhang continous MRS parameter estimation as applied on DNA methylation data generated with Ion AmpliSeq and SureSelect technology. Data were transformed using the following equation: MRS Cont. HTS Transformed = − 1.72 + 0.61*MRS Cont. HTS. Fig. S7 Scatterplot of PoAm and chronological age correlation (R=0.024) after applying data transformation. Fig. S8 Scatterplot of the correlation between MRS and chronological age (R=0.783) after data transformation, taking into account the continuous (A) and categorical (B) character of the MRS parameter. Fig. S9 Analysis of the impact of the applied thres [file 13148_2023_1545_MOESM2_ESM.pdf]

Supplementary Materials

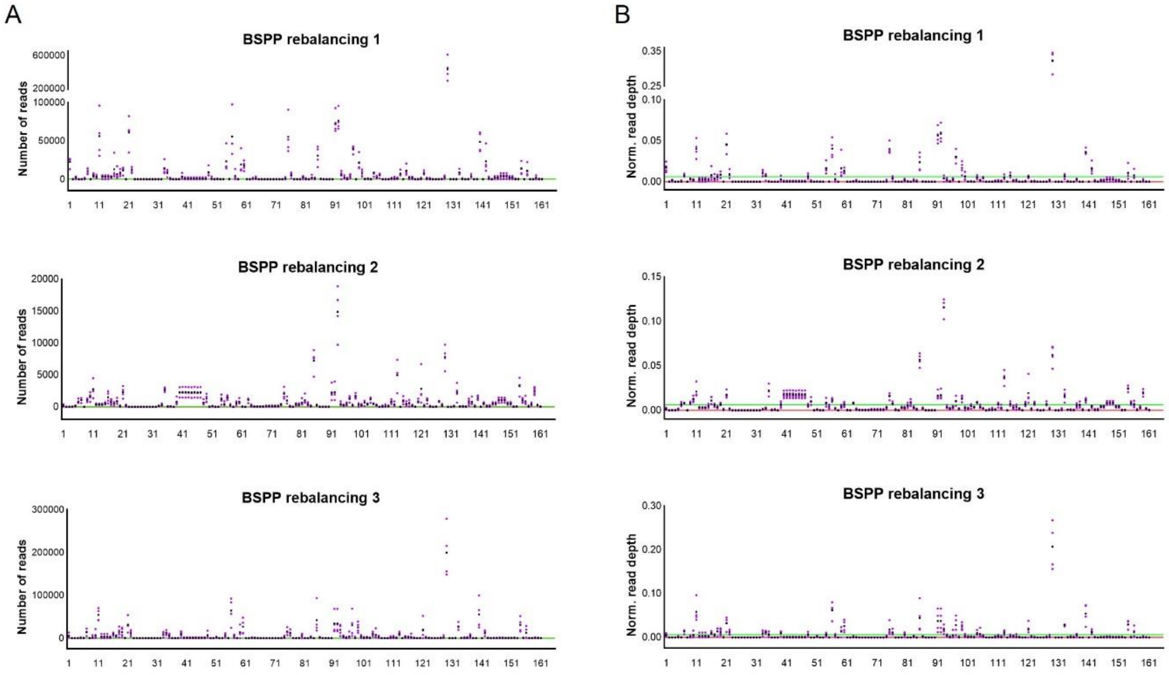

Fig. S1 *Raw reads number distribution (A) and normalized read depth (B) for three sequencing runs with three approaches to rebalance the probes performed for the BSPP technology.*

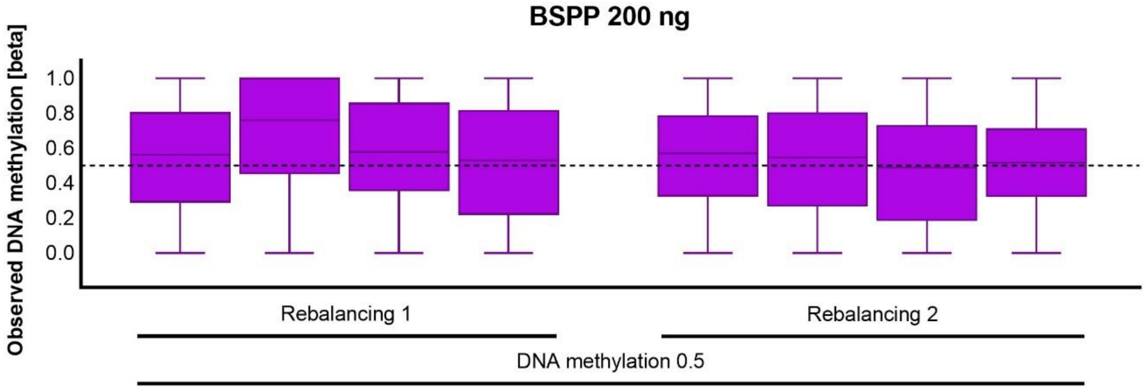

Fig. S2 *Accuracy of DNA methylation measurement for the BSPP technology. Libraries were prepared for 0.5 DNA methylation standards.*

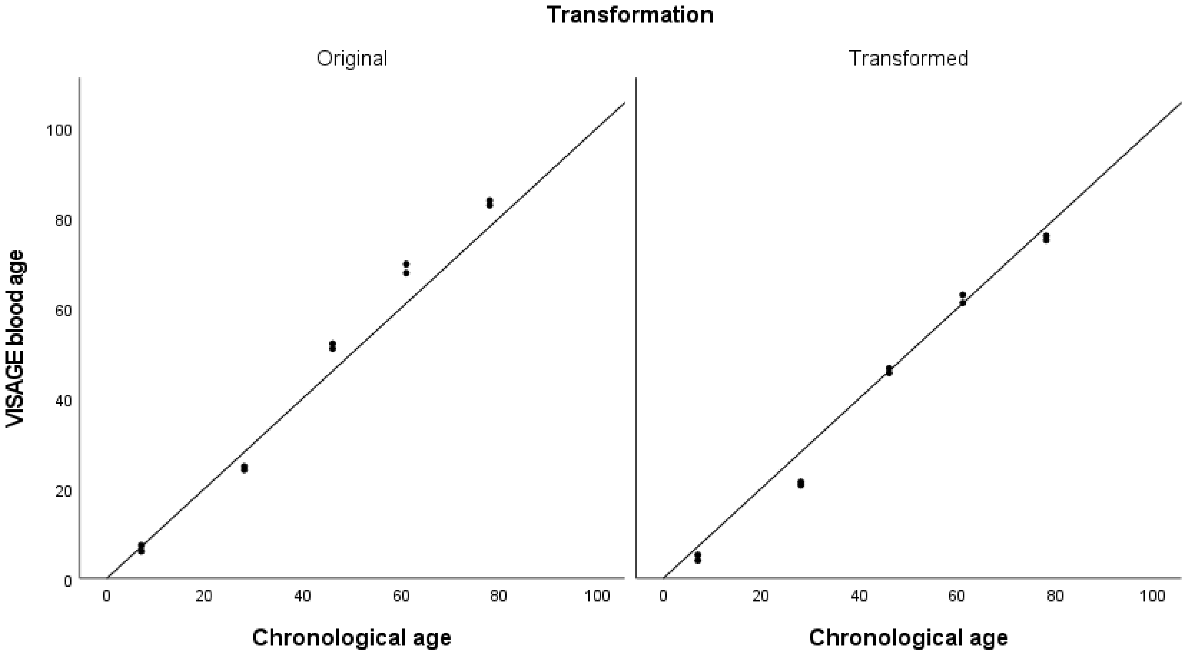

Fig. S3 *Data transformation and impact on the accuracy of age prediction using the original VISAGE blood age model trained on Illumina sequencing data as applied on DNA methylation data generated with Ion AmpliSeq technology. Data were transformed using the following equation: VISAGE blood age Ion AmpliSeq TRANSFORMED = -1.61 + (VISAGE blood age Ion AmpliSeq*0.93).*

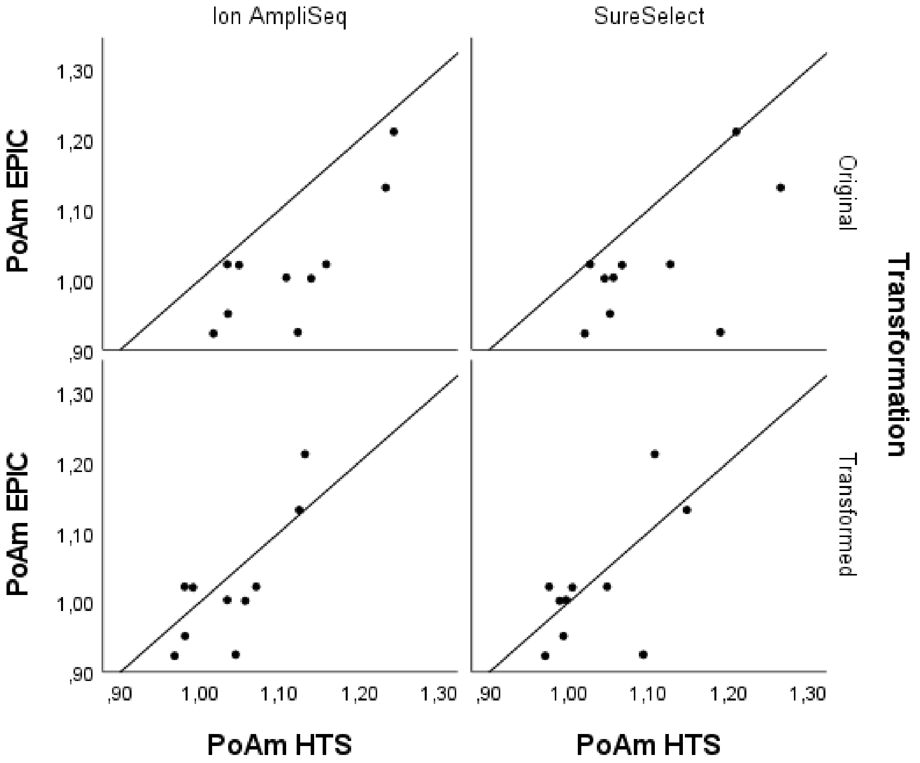

Fig. S4 *Data transformation and impact on the accuracy of PoAm parameter estimation as applied on DNA methylation data generated with Ion AmpliSeq and SureSelect technology. Data were transformed using the following equation: PoAm HTS Transformed = 0.23 + (PoAm HTS*0.72).*

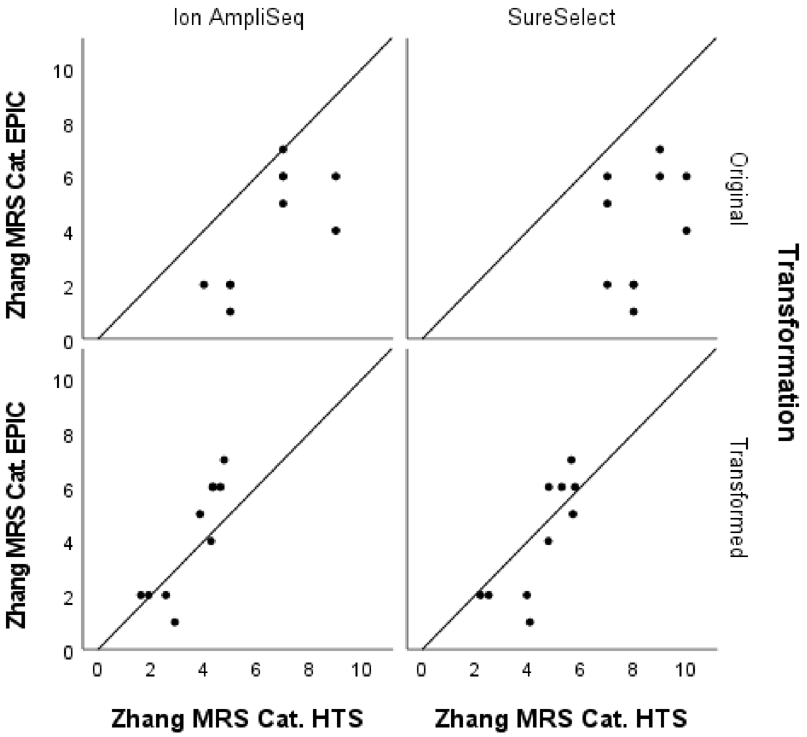

Fig. S5 *Data transformation and impact on the accuracy of Zhang categorical MRS parameter estimation as applied on DNA methylation data generated with Ion AmpliSeq and SureSelect technology. Data were transformed using the following equation: MRS Cat. HTS Transformed = 25.76 + 13.29*MRS Cont. HTS Transformed + 1.81*MRS Cont. HTS Transformed*MRS Cont. HTS Transformed.*

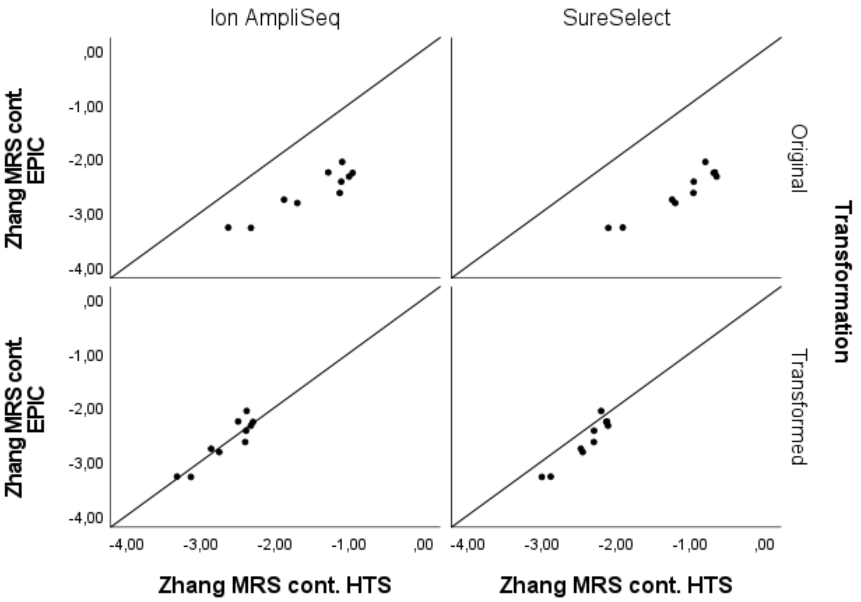

Fig. S6 *Data transformation and impact on the accuracy of Zhang continous MRS parameter estimation as applied on DNA methylation data generated with Ion AmpliSeq and SureSelect technology. Data were transformed using the following equation: MRS Cont. HTS Transformed = -1.72 + 0.61*MRS Cont. HTS.*

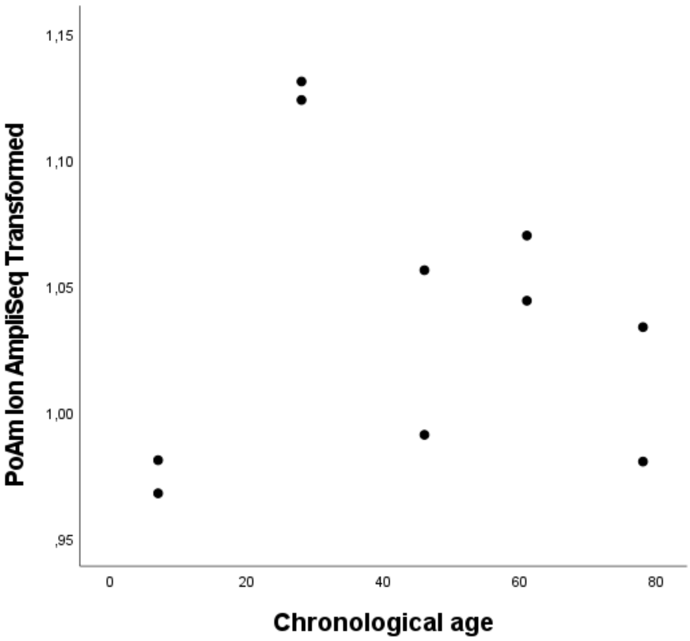

Fig. S7 *Scatterplot of PoAm and chronological age correlation (R=0.024) after applying data transformation.*

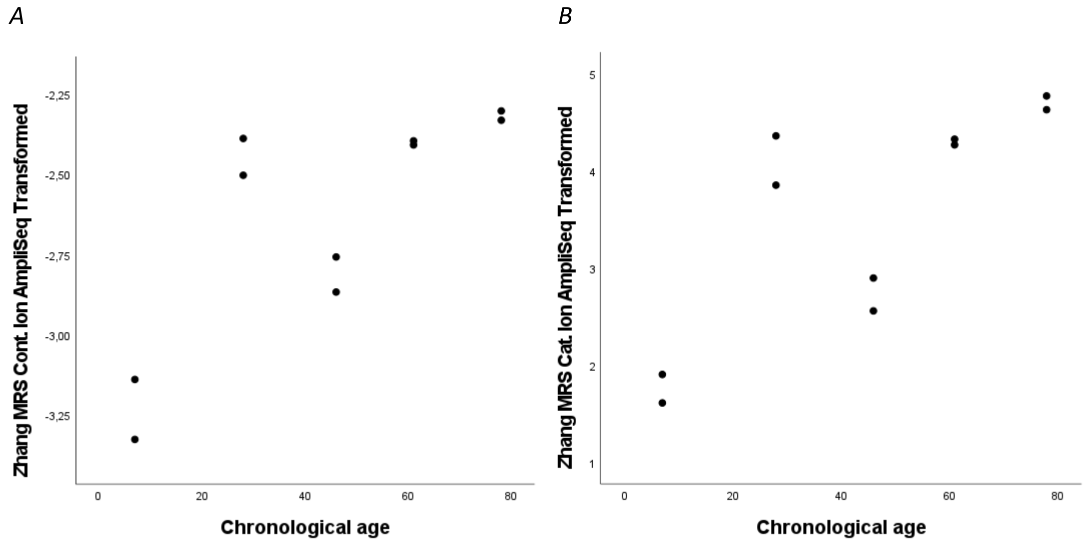

Fig. S8 *Scatterplot of the correlation between MRS and chronological age (R=0.783) after data transformation, taking into account the continuous (A) and categorical (B) character of the MRS parameter.*

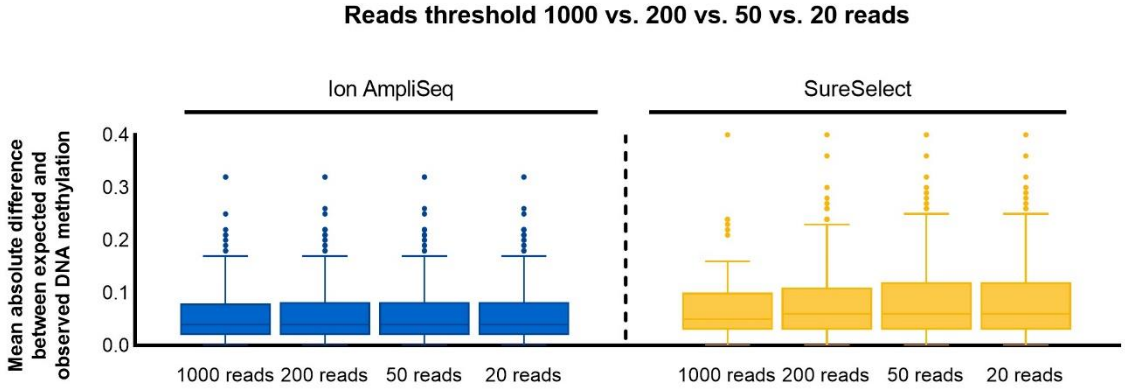

Fig. S9 *Analysis of the impact of the applied threshold of the minimum number of reads on the precision of DNA methylation determination: mean absolute difference between the observed and expected DNA methylation beta values and the standard deviation of the results.*

***Table S2** Cytosines reaching the requested read depth threshold for the technologies tested.*

**Number of cytosine targets**					
**Read depth threshold**	**1x**	**20x**	**50x**	**200x**	**1000x**
Ion AmpliSeq*	155/155 (100%)	155/155 (100%)	155/155 (100%)	154/155 (99.4%)	127/155 (81.9%)
SureSelect*	160/161 (99.4%)	158/161 (98.1%)	156/161 (96.9%)	135/161 (83.9%)	76/161 (47.2%)
BSPP Exp1**	55/161 (34.2%)	53/161 (32.9%)	45/161 (28.0%)	44/161 (27.3%)	38/161 (23.6%)
BSPP Exp2 **	118/161 (73.3%)	100/161 (62.1%)	93/161 (57.8%)	69/161 (42.9%)	23/161 (14.3%)
BSPP Exp3**	44/161 (27.3%)	39/161 (24.2%)	38/161 (23.6%)	36/161 (22.4%)	31/161 (19.3%)

*Results achieved across four samples sequenced on one selected sequencing run (for DNA input optimal for the technology)

**Results achieved for three different experiments and sequencing runs, for different conditions of probe panel optimisation

***Table S3** Pearson correlation analysis for different age-related parameters and chronological age, using data generated with Ion AmpliSeq technology.*

**Correlations**		**VISAGE**	**Hannum age**	**PoAm**	**Zhang MRS**	**Age**
**VISAGE age**	Pearson R	1	0.992**	-0.028	0.733*	0.995**
	Sig. (2-tailed)	NA	**1.77E-08**	0.938	0.016	**2.88E-09**
	N	10	10	10	10	10
**Hannum age**	Pearson R	0.992**	1	0.078	0.788**	0.995**
	Sig. (2-tailed)	**1.77E-08**	NA	0.831	**0.007**	**3.03E-09**
	N	10	10	10	10	10
**PoAm**	Pearson R	-0.028	0.078	1	0.564	0.024
	Sig. (2-tailed)	0.938	0.831	NA	0.090	0.947
	N	10	10	10	10	10
**Zhang MRS**	Pearson R	0.733*	0.788**	0.564	1	0.783**
	Sig. (2-tailed)	**0.016**	**0.007**	0.090	NA	**0.007**
	N	10	10	10	10	10
**Age**	Pearson R	0.995**	0.995**	0.024	0.783**	1
	Sig. (2-tailed)	**2.88E-09**	**3.03E-09**	0.947	**0.007**	NA
	N	10	10	10	10	10
**. Correlation is significant at the 0.01 level (2-tailed).						
*. Correlation is significant at the 0.05 level (2-tailed).						

Table S4 *Pearson correlation analysis of results obtained for individual clocks with different DNA methylation data collection technologies.*

Correlations			
**Hannum EPIC**		**Hannum AmpliSeq**	**Hannum SureSelect**
	Pearson R	0.996	0.998
	Sig. (2-tailed)	**1.58E-09**	**5.90E-11**
**VISAGE EPIC**		**VISAGE AmpliSeq**	**VISAGE SureSelect**
	Pearson R	0.994	0.991
	Sig. (2-tailed)	**4.41E-09**	**3.32E-08**
**PoAm**		**PoAm AmpliSeq**	**PoAm SureSelect**
	Pearson R	0.781	0.619
	Sig. (2-tailed)	**0.008**	0.057
**Zhang MRS**		**Zhang AmpliSeq**	**Zhang SureSelect**
	Pearson R	0.921	0.950
	Sig. (2-tailed)	**0.00015**	**0.000026**

Supplementary Methods

Bisulfite padlock probes protocol

Bisulfite conversion of DNA samples with DNA input at the level of 500 and 250 ng was done using the EZ DNA Methylation Direct Kit (Zymo Research) according to the manufacturer’s guidelines. 10 µl of the converted DNA was used for probe hybridization following the library-free protocol described in [1]. Hybridization was performed overnight under the following conditions: 94˚C for 30 s, gradually decreasing temperature to 55˚C at 0.1˚C/s and 55˚C for 20 h. Gap-filling and ligation master mix enriched in 1.67 mM NAD^+ was added to the samples held at 55°C for a further 20 hours followed by 2 minutes at 94°C. The purified products were amplified using Qiagen Multiplex PCR Master Mix (Qiagen) and dual indexing adapters. The PCR program was: 95°C for 15 min, 38 cycles of [94°C x 30 s, 58°C x 90 s, 72°C x 60 s] and 60°C for 15 min followed by a hold at 4°C. After size selection by cutting the bands from the agarose gel, the barcoded libraries were purified using the QIAquick Gel Extraction Kit (Qiagen) and evaluated using the High Sensitivity DNA Kit (2100 Bioanalyzer instrument, Agilent). Libraries were prepared for sequencing according to the MiSeq System Denature and Dilute Libraries Guide, Protocol A, including library concentration 5 pM with the addition of 10% PhiX. Four samples were processed together and sequenced per one flowcell of the MiSeq Reagent Kit v3 (2x75 bp) using the MiSeq FGx System. Sequencing reads in fastq files were pre-evaluated using FastQC software, then mapped to the bisulfite-converted GRCh38 genome with Bismark 0.19.0 and subjected to methylation calling according to the procedure described for SureSelect technology.

1. Diep D, Plongthongkum N, Gore A, Fung HL, Shoemaker R, Zhang K. Library-free methylation sequencing with bisulfite padlock probes. Nat Methods. 2012;9:270–2.
